# Hormonal and Glycemic Responses During and After Constant- and Alternating-Intensity Exercise

**DOI:** 10.3390/jcm14020457

**Published:** 2025-01-13

**Authors:** Sofia Spanoudaki, Maria Maridaki, Costas Chryssanthopoulos, Anastassios Philippou

**Affiliations:** 1 Faculty of Physical Education and Sport Science, National and Kapodistrian University of Athens, 41 Ethnikis Antistasis, 17237 Dafni, Greece; sspanoud@yahoo.gr (S.S.); mmarida@phed.uoa.gr (M.M.); 2 Medical School, National and Kapodistrian University of Athens, 75 Mikras Asias, 11527 Goudi, Greece; chryssan@phed.uoa.gr

**Keywords:** alternating intensity, constant exercise, glucose metabolism, insulin, leptin, prolactin

## Abstract

**Background/Objectives:** Glucose metabolism and hormonal responses are largely affected by exercise intensity, which exhibits an alternating pattern in many sports activities. The aim of this study was to investigate and compare glycemic and hormonal responses during and after exercise of constant (CON) and alternating (ALT) intensity with the same duration and total work. **Methods:** Ten healthy male volunteers performed two, 60 min cycling exercise bouts in random order: the ALT bout, where exercise intensity alternated between 46.5 ± 1.9% of VO_2_max for 40 s and 120% of VO_2_max for 20 s, so the mean intensity was at 105% of the lactate threshold (LT), and the CON exercise bout, where the intensity was constant at 105% of LT (70 ± 4.7% of VO_2_max). **Results:** No significant differences were observed in blood glucose concentrations between the two exercise protocols (*p* = 0.22) or over time at any time point measured, i.e., before, at 30 and 60 min of each exercise bout, and 60 min post-exercise (*p* > 0.05). Circulating insulin levels decreased significantly over time in both protocols (*p* < 0.01 and *p* < 0.05 in ALT and CON, respectively); nevertheless, they did not differ between the exercise protocols (*p* = 0.45). Similarly, there were no significant differences in serum leptin and prolactin levels between the two protocols (*p* = 0.77 and *p* = 0.80 in ALT and CON, respectively); however, circulating levels of leptin decreased at 30 and 60 min of exercise only in CON (*p* < 0.05) and those of prolactin at 60 min of exercise only in ALT (*p* < 0.05) compared to pre-exercise values. **Conclusions:** Cycling exercise of constant or alternating moderate intensity (~70% of VO_2_max) with the same duration induces similar glycemic but differential over time hormonal responses in healthy males.

## 1. Introduction

It is well established that regular physical activity reduces the risk of cardiovascular and metabolic diseases and positively influences important functional and clinical outcomes of these patients [1,2]. Guidelines from the American College of Sports Medicine (ACSM) recommend that adults should accumulate at least 150 min of moderate to vigorous exercise per week to gain cardiometabolic health benefits [3]. Endurance exercise training can be performed either with continuous aerobic training (CON; high-volume steady-intensity exercise, lasting for more than 20 min) or with high-intensity interval training (HIIT). HIIT consists of short bursts (from 6 s to 4 min) of vigorous exercise (equal to or higher than 90% of VO_2_max) interrupted by periods of less intense exercise or complete rest. The rationale of HIIT is to accumulate exercise at an intensity greater than 90% of VO_2_max for a shorter total time compared to continuous exercise [4]. HIIT is a time-efficient training that induces rapid adaptations in skeletal muscle and physical performance compared to CON; however, both types of exercise are associated with improved metabolic regulation and health markers [4,5,6,7].

Exercise is a stress stimulus affecting homeostatic mechanisms and hormonal responses, depending on the type, intensity, and duration of exercise [8]. Several studies have described the hormonal acute responses and adaptations to different types of exercise and training [8,9,10,11].

Glucose is a major fuel source for contracting muscles and glucose uptake increases instantly during exercise [12]. Muscle contraction–stimulated glucose uptake is the main mechanism responsible for glycemic regulation during moderate exercise and can occur despite decreased levels of insulin [13,14,15]. Exercise increases the sensitivity of skeletal muscle to the action of insulin, which may counterbalance blood insulin levels [16]. The reduction in circulating insulin during exercise is critical not only for physical performance but also for preventing fatigue due to early hypoglycemia [17]. The effects of exercise on blood glucose and insulin levels are dependent on several factors, such as carbohydrate consumption, exercise type, duration and intensity, and individual’s physical conditioning [18].

Leptin is related to glucose and free fatty acids (FFAs) metabolism as well as to health status. Blood leptin levels are reduced acutely during fasting or with energy restriction, while they increase with refeeding [19]. Exercise alters the energy balance, and thus, it might be expected to change circulating leptin levels. Blood leptin has been measured after a single bout of (maximal or submaximal) exercise of short or long duration in humans, and the reported exercise-induced leptin responses have been conflicting [20,21]. Specifically, it was shown that serum leptin levels do not alter after a single-bout non-exhaustive exercise of short duration, while they were found to drop when the duration of exercise was 60 min or longer [21]. Moreover, the intensity of exercise may also affect the circulating leptin levels [22].

Leptin and several adipokines, including adiponectin, are modulated by prolactin, affecting adipose tissue and peripheral insulin sensitivity [23]. Elevated prolactin levels have been associated with a compensatory increase in insulin response to hyperglycemia and are also associated with improved insulin sensitivity [24,25]. There are studies showing that both moderate aerobic exercise and short-term HIIT may induce acute responses and rapid adaptations of the hypothalamic–pituitary–adrenal (HPA) axis, affecting prolactin levels [9,11]. Prolactin increases proportionally to the intensity of exercise and the magnitude of its increase may be augmented with longer exercise duration. Particularly in males, plasma prolactin increases during exercise in relation to exercise intensity and duration [26]. Overall, the effect of submaximal exercise on prolactin appears controversial showing an increase [27], decrease, or no change [28,29].

In this study, we monitored the blood glucose and hormonal responses (insulin, leptin, and prolactin) during cycling exercise and the recovery period of constant- and alternating intensity, though with the same duration and total work, in healthy individuals. We posed the hypothesis that the circulating levels of glucose and the aforementioned hormones would be differently altered over time in the two exercise protocols, thus suggesting potentially differential, intensity-dependent regulatory mechanisms.

## 2. Materials and Methods

### 2.1. Participants

Ten healthy, moderately trained young men volunteered to participate in the study. The anthropometric characteristics of the participants are shown in Table 1. The study was performed in accordance with the ethical standards of the Declaration of Helsinki for experimentation with human subjects. All participants were informed of all procedures and purposes of the study and gave their written informed consent.

### 2.2. Experimental Design

Each participant completed three preliminary tests (i.e., familiarization, submaximal, and maximal VO_2_ (VO_2_max)) and two main trials in a random order, i.e., constant (CON)- and alternating (ALT)-intensity exercise. Both CON and ALT trials had 60 min duration and were performed after a 10 min warm-up period at the same time of the day and with a time interval of 7 days apart. In the ALT, the exercise intensity alternated between about 45% of VO_2_max for 40 s and 120% of VO_2_max for 20 s, so the mean intensity was at 105% of the lactate threshold (LT), while in CON, the intensity was constant at 105% of LT. All tests were conducted on the same modified friction-loaded cycle ergometer (Monark model 824E, Varberg, Sweden), in which a special modification was made to run the alternating-intensity exercise test [30,31].

### 2.3. Experimental Procedures

After being fully informed about experimental procedures and giving written consent, anthropometric measurements were performed. Percent of body fatness was estimated by the skinfold method (biceps, triceps, suprailiac, subscapular, abdominal, anterior thigh, and medial calf) using appropriate equations [32].

After the completion of the anthropometric measurements, participants performed the maximal VO_2_ test. The VO_2_max was determined by a continuous incremental cycling exercise protocol to exhaustion, using an open circuit—spirometry (MedGraphicsCPX/D, Saint Paul, MN, USA). VO_2_max was defined as the highest VO_2_ obtained in 30 s. Prior to each test, the gas analyzers were calibrated, using gases of known O_2_ and CO_2_ concentrations. Maximal power output (POmax_)_ was defined as the highest average power output during the last minute of the VO_2_max protocol.

After three days and no longer than a week, a second, submaximal test was conducted to determine the blood lactate threshold (LT). Specifically, each participant performed 5 submaximal cycling sets, each one lasting for 5 min at a frequency of 70 rpm, using the cycle ergometer. A digital display adjusted to the cycle ergometer was used to monitor the pedaling frequency. For the LT determination, 20 μL of blood was drawn from the participant’s fingertip, diluted with 200 μL percloric acid, and stored at −20 °C until analysis. LT was obtained from non-linear plots of exercise intensity (power) against lactate concentration based on the 4 mmole/L method [33].

The third preliminary test included the familiarization with the two main trials, i.e., the CON and ALT exercise protocols. Furthermore, participants were asked to abstain from consuming food, coffee, and alcohol 4 h before each preliminary test. Lastly, the two main trials were conducted 1 week apart in random order at the same time of the day. Participants were fasted overnight and abstained from exercise 2 days prior to the main trials. Also, they were asked to record their diet the day before the first main trial and replicate this the day before the second main trial.

### 2.4. Constant- and Alternating-Intensity Protocol

Each protocol lasted for 1 h at a mean intensity of 105% of the LT, which is 70% of VO_2_max, as previously described [30,31]. The work rate of 70% of VO_2_max was chosen since it is considered moderate-intensity exercise, slightly above the lactate threshold, and could be sustained for a prolonged period. Briefly, during the CON exercise, participants were cycling at a power output corresponding to 105% of the LT (154.3 ± 17.3 W, 57.8% = ± 6.5% of power max or 69.8 ± 4.7% VO_2_max). During ALT exercise, 40 s light cycling exercise (46.5 ± 1.9% VO_2_max) was alternated with 20 s intervals of intense cycling at 120% of VO_2_max (158.8 ± 20.5 W, 59.4 ± 7.8% of power max). The mean intensity (power output) was the same in both CON and ALT protocols.

### 2.5. Blood Sampling and Analyses

Blood samples were withdrawn from an antecubital vein of each participant at the following time points: prior to the test, during the last seconds of 30 and 60 min of each exercise bout, and 60 min post-exercise, at the same time of the day for all subjects. The subjects were seated, and 10 mL of blood were drawn in EDTA-containing tubes. Plasma was collected after centrifugation at 4000 rpm for 10 min at 4 °C, stored frozen in 0.5 mL aliquots at −80 °C, and only thawed once for analysis. For serum analyses, blood samples were allowed to clot at room temperature for 30 min, and again serum was collected after centrifugation at 4000 rpm for 10 min at 4 °C, stored frozen in 0.5 mL aliquots at −80 °C, and only thawed once for analysis. Hematocrit and hemoglobin were measured via routine methods (Abbott, IL, USA), and exercise-induced changes in plasma volume were calculated and corrected using hematocrit and hemoglobin changes according to Dill and Costil equations [34].

Serum glucose (GL), insulin (INS), prolactin (PRL), and leptin (LE) concentrations were determined by standard sandwich enzyme-linked immunosorbent assay (ELISA) protocols using commercially available kits (leptin serum KAP2281, KAP1441 (PR)BioSource International Inc., Camarillo, CA, USA) according to manufacturer’s instructions. The color formation was measured by a microplate reader (Versamax, Molecular Devices, San Jose, CA, USA) at 450 nm, and calculations were carried out using SoftMax Pro 7 software (Molecular Devices, San Jose, CA, USA). All samples were run simultaneously and analyzed in duplicate, and the results were averaged. According to the manufacturers, the minimal detection limits of the assays used were 0.93 pg mL^−1^, 56.72 pg mL^−1^, 0.05 ng mL^−1^, 0.12 ng/mL, 0.05 μIU mL^−1^, 0.054 ng dL^−1^, 1 ng mL^−1^, 83 pg mL^−1^, 3.8 pg mL^−1^ for GL, INS, PRL, and LE, respectively, while the intra- and inter-assay coefficient of variation (CV) were as follows: 4.0% to 4.2% and 1.9% to 6.4% for GL, 7.3% to 10.5% and 7.8% to 13.4% for INS, 2.3 % to 5.0% and 5.4% to 8.0% for PRL, and 1.8% to 2.1% and 8.2% to 10.8% for LE.

The homeostasis model assessment (HOMA) was also assessed. This index allows for the quantitative assessment of insulin action by using fasting concentrations of glucose and insulin according to the formula: HOMA = (FPI × FPG)/22.5, where FPI and FPG are the fasting insulin in μU/mL and glucose concentration in mmol/L, respectively. The formula is based on an array of predicted glucose and insulin values that would be expected for many potential combinations of insulin actions [35].

### 2.6. Statistical Analysis

A two-way analysis of variance (ANOVA) with repeated measures over time was used to evaluate differences between the two exercise conditions in all measurements (SPSS v. 25 statistical package). A non-parametric (Friedman) test was conducted where the data had violated the assumptions necessary to run the repeated measures one-way ANOVA (e.g., data not normally distributed). Where a significant F ratio was found for the main effect (*p* < 0.05), the means were compared using Dunnett’s post hoc test, or Wilcoxon signed-rank test with Bonferroni correction for non-parametric tests. Pearson’s correlation coefficient (r) was used to determine correlations between variables. Data from preliminary tests are presented as means ± SD, while data from GL, INS, PRL, and LE are presented as mean ± standard error of the mean (SE). The level of statistical significance was set at *p* < 0.05.

## 3. Results

### 3.1. Glucose Responses to ALT and CON Exercise

**Figure 1:**
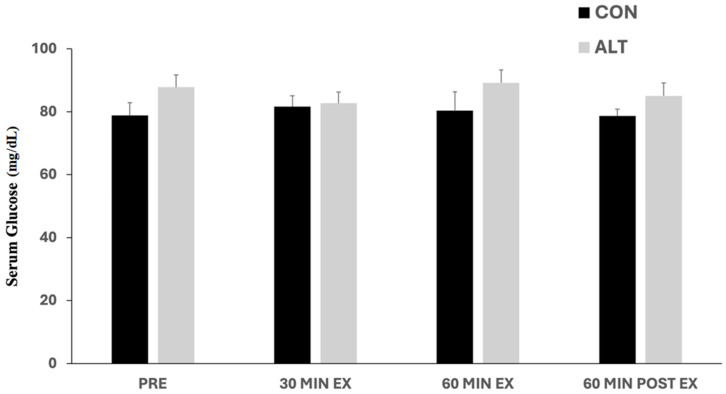
Serum glucose concentrations before (Pre), in response to constant (CON)- and alternating (ALT)-intensity exercise (at 30 and 60 min), and 60 min post-exercise. No significant changes were observed in glucose levels over time compared to baseline (Pre) or between the exercise bouts. Values are presented as mean ± standard error of the mean (mean ± SE).

The serum glucose acute responses to ALT and CON exercise are shown in Figure 1. Glucose levels were slightly changed during exercise in both protocols, though without significant differences (*p* > 0.05) over time at any time point measured. Also, there was no difference in glucose responses between the two exercise protocols (main effect for the group; *p* = 0.22). Considerable inter-individual variations in glucose levels were observed between subjects (numeric data are provided in Supplementary Table S1).

### 3.2. Insulin Responses to ALT and CON Exercise

**Figure 2:**
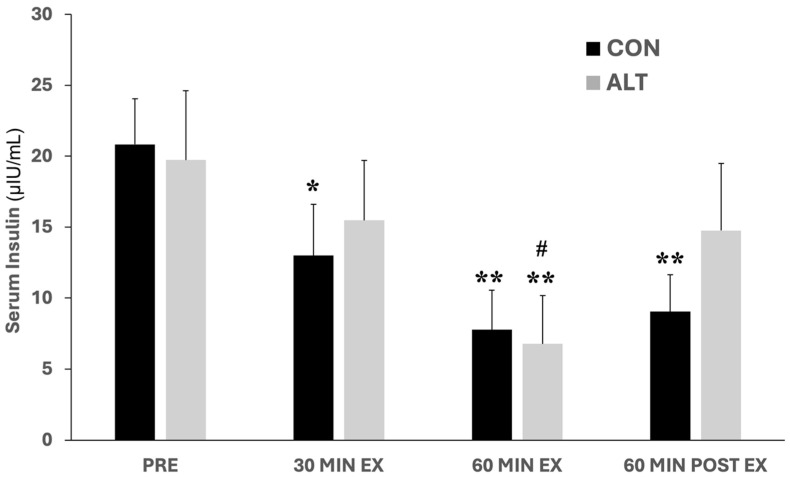
Serum insulin concentrations at baseline (Pre), in response to constant (CON)- and alternating (ALT)-intensity exercise (at 30 and 60 min), and 60 min post-exercise. Insulin levels were gradually decreased at 30 and 60 min of CON exercise and remained significantly lower by 60 min post-exercise compared to baseline. In the ALT exercise protocol, insulin levels decreased significantly only at 60 min of exercise compared to baseline. Values are presented as mean ± standard error of the mean (mean ± SE); significantly different compared to baseline: * *p* < 0.05; ** *p* < 0.01. Significantly different compared to 30 min of exercise and 60 min post-exercise: ^#^ *p* < 0.05.

The serum insulin data are presented in Figure 2. Circulating insulin levels decreased during CON exercise at all time points measured compared to baseline. Specifically, in CON, significant differences were observed between the baseline levels and at 30 (*p* < 0.05) and 60 min (*p* < 0.01) of exercise, as well as at 60 min post-exercise (*p* < 0.01). However, during ALT exercise, a significant difference compared to baseline was only observed at 60 min of exercise (*p* < 0.01), while insulin levels were significantly lower at 60 min compared to 30 min of exercise and 60 min post-exercise (*p* < 0.05, Figure 2; numeric data are provided in Supplementary Table S1)

No significant differences were observed in the insulin levels between the two exercise protocols (main effect for the group; *p* = 0.45). Also, there was no difference in the HOMA index between the two protocols (HOMA CON: 4.06 ± 1.99 vs. HOMA ALT: 4.27 ± 2.85; *p* > 0.05).

### 3.3. Leptin Responses to ALT and CON Exercise

**Figure 3:**
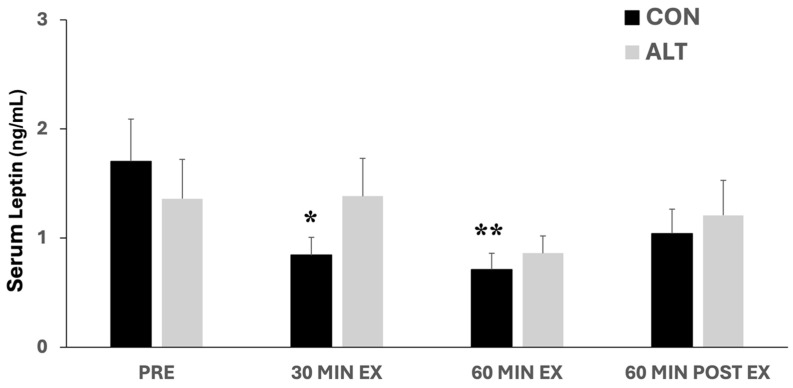
Serum leptin concentrations at baseline (Pre), in response to constant (CON)- and alternating (ALT)-intensity exercise (at 30 and 60 min), and 60 min post-exercise. Leptin levels were gradually decreased at 30 and 60 min of CON exercise only, compared to baseline. Values are presented as mean ± standard error of the mean (mean ± SE). Significantly different compared to baseline: * *p* < 0.05; ** *p* < 0.01.

Leptin responses to the different exercise protocols are shown in Figure 3. There were no significant differences between the two protocols (main effect for the group: *p* = 0.77) or over time (main effect for time: *p* = 0.2). Interestingly, however, only in the CON exercise protocol, circulating leptin levels decreased significantly at 30 min and 60 min of exercise compared to baseline (Pre) (*p* < 0.05–0.01, Figure 3; numeric data are provided in Supplementary Table S1).

### 3.4. Prolactin Responses to ALT and CON Exercise

**Figure 4:**
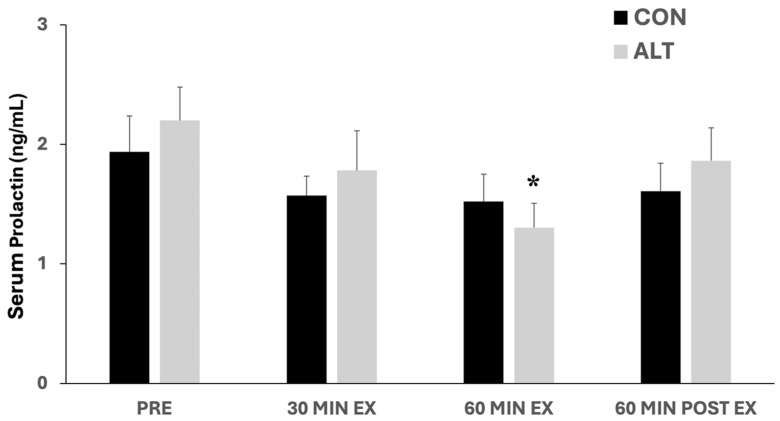
Serum prolactin concentrations at baseline (Pre), in response to constant (CON)- and alternating (ALT)-intensity exercise (at 30 and 60 min), and 60 min post-exercise. Prolactin levels were significantly decreased only at 60 min of ALT exercise compared to baseline. Values are presented as mean ± standard error of the mean (mean ± SE). Significantly different compared to baseline: * *p* < 0.05.

Serum prolactin responses to acute CON and ALT exercise are shown in Figure 4. There was no significant difference between the two exercise protocols (main effect for the group: *p* = 0.80) or over time (main effect for time: *p* = 0.21). However, only in the ALT exercise protocol, prolactin levels significantly decreased (*p* < 0.05) at 60 min of exercise compared to baseline (Pre). Numeric data are provided in Supplementary Table S1.

## 4. Discussion

In this study, we examined and compared the glycemic and hormonal responses between CON and ALT exercise at the same mean intensity lasting for 60 min in healthy participants, to reveal a potential differential regulation of these responses depending on exercise intensity variations. We found no differences in serum glucose, insulin, leptin, and prolactin concentrations between the two types of exercise. Interestingly, however, different over time hormonal responses were observed; insulin levels gradually decreased at 30 and 60 min of CON exercise and remained significantly lower 60 min post-exercise compared to baseline, while in ALT protocol, insulin levels decreased only at 60 min of exercise compared to baseline. Moreover, the serum concentration of leptin decreased over time only in the CON exercise protocol and that of PRL only in the ALT protocol, while all hormones exhibited the largest changes at 60 min of exercise.

During exercise of moderate intensity, the main mechanism responsible for regulating blood glucose is muscle contraction, which stimulates glucose uptake that occurs despite decreased levels of insulin [13,36], and exercise intensity might influence the mechanism(s) that induces these responses [37]. In this study, both exercise protocols were performed at the same mean intensity, i.e., at 105% of LT, with the same duration and total work; thus, despite the different modes of cycling exercise (CON vs. ALT), the glycemic responses appeared to be regulated by mean intensity rather than the peak intensity or the intensity fluctuations during exercise. Similarly to our findings, other studies found that blood glucose levels did not change after 90 min of moderate exercise at 70% of VO_2_max in healthy adults [17], or during and after 30 min of HIIT or moderate-intensity continuous exercise in patients with type II diabetes [38].

Nevertheless, Peake et al. [39] compared the effects of HIIT versus work-matched moderate-intensity continuous exercise on metabolism and counterregulatory stress hormones. They found that blood glucose increased after both types of exercise, though HIIT exercise resulted in higher glucose and lactate compared to moderate-intensity continuous exercise. However, 1 h post-exercise, glucose levels returned to pre-exercise values and insulin decreased during recovery in both protocols. The differences in glycemic responses between those two protocols and their inconsistency with our findings might be attributed to higher physiological stress induced by the HIIT protocol compared to the continuous moderate-intensity protocol, as reflected by the higher levels of lactate and the counterregulatory hormone norepinephrine in HIIT found in the study of Peake et al. [39] as catecholamines have been implicated as potent stimulators of hepatic glucose release during exercise to avoid hypoglycemia [40,41,42]. The ALT and CON protocols used in our study resulted in similar responses regarding the lactate concentration, epinephrine, and growth hormone levels, while, interestingly, norepinephrine exhibited higher values in CON compared to ALT exercise [31].

During moderate exercise (e.g., at 60% of VO_2_max) in healthy individuals, increased glucose uptake by muscles is counterbalanced by an equal increase in liver glucose output; thus, blood glucose levels remain unchanged. Concurrently, there is a reduction in insulin levels, which increases hepatic sensitivity to glucagon action, increasing glucose production [13]. In addition, elevated catecholamine levels result in suppression of insulin and enhancement of glucagon to increase gluconeogenesis (GNG) [43], while lactate availability increases the rate of GNG during exercise at an intensity close to the LT [44]. Moreover, maintenance of blood glucose during submaximal exercise (e.g., 30 min up to an hour at 40–70% of VO_2_max) is regulated mainly by the insulin/glucagon response [45]. In our study, serum glucose remained unchanged in both protocols, and the HOMA index did not differ between them, which is in line with the findings of previous studies [13,16,43,44,45,46]. Interestingly, though, insulin concentration significantly decreased over time mainly during and after the CON protocol. More specifically, in this study, insulin tended to return to baseline after a transient decrease at 60 min of ALT exercise, indicating its differential regulation compared to the earlier and persistent decrease during and after the CON exercise (Figure 2). A decrease in circulating insulin during exercise is considered a major advantage for the working muscles that need less insulin for glucose uptake [47], while insulin appears not to play a major role in the glycemic responses during recovery [13].

Previous studies on the hormonal responses to short-term exercise (<60 min) showed that this exercise, regardless of its intensity, did not alter the circulating leptin levels [21,48,49,50]. Nevertheless, others have reported that both HIIT and CON exercise (30 min at 50% of peak velocity) resulted in a significant decrement in leptin concentration, without differences between the two types of exercise [51]. After a long-term exercise (≥60 min), alterations in leptin concentration have been reported and attributed to other hormonal changes, stimulation of free fatty acids (FFAs) metabolism, or exercise-induced energy expenditure higher than 800 kcal [20,21]. Garcia et al. [52] suggested that physical fitness levels regulate leptin-induced fat oxidation during exercise, while this response is affected by gender. Specifically, cardiorespiratory fitness can modulate leptin’s signaling pathway in skeletal muscles, while higher levels of leptin are associated with higher levels of insulin and, consequently, with insulin resistance, due to a break in the insulin signaling pathway caused by hyperinsulinemia [52]. Circulating leptin is associated with fat oxidation and insulin resistance, and women respond differently compared to men due to the larger adipose tissue, higher body fat/skeletal muscle ratio, and the higher production rate of leptin per unit mass of adipose tissue [52,53].

Although in this study we did not examine energy expenditure and FFA release during the exercise protocols, however, respiratory quotient (RQ) during them (data have been reported in a previous publication [31]) was around 1, indicating mainly glucose oxidation. Zaccaria et al. [54] evaluated the effect of exercise duration and its corresponding energy expenditure on leptin levels after 4 h treadmill exercise at 65% of VO_2_max. They found that leptin levels decreased at the end of exercise, while glucose did not change throughout the exercise bout. Their findings suggested that during prolonged, moderate-intensity exercise, leptin decrement might be associated with total energy expenditure and, also, with norepinephrine levels, which seems to play an important role in the inhibition of leptin secretion [55].

Interestingly, the exercise protocols used in this study have been shown to result in differential norepinephrine responses, with higher values of norepinephrine being observed in CON compared to ALT exercise [31]. Correspondingly, we found a significant reduction in serum leptin concentrations at 30 and 60 min of CON exercise only compared to baseline. Overall, these findings might suggest that the differences in leptin responses over time between the CON and ALT are likely related to the higher levels of norepinephrine during CON exercise and its effects as a potent inhibitor of leptin secretion [55].

Lastly, in this study, we explored the potential responses of prolactin, which has been found to exhibit conflicting responses to exercise, depending on its duration and intensity [26,28]. We found only a modest and transient decrease in circulating prolactin levels, interestingly, only in ALT exercise. Plasma prolactin levels were previously found to decrease during submaximal work [26]. Specifically, 1 h of submaximal work below the lactate threshold was accompanied by a slight decrease in plasma prolactin levels. However, when the participants exercised up to maximal intensity and exhaustion, a sharp rise in prolactin occurred when the anaerobic threshold (AT) was reached and continued to increase after stopping the exercise [26]. An intensity-dependent stimulation of prolactin has been also reported by others [56,57], showing that prolactin concentrations increased only after exercise reached an intensity of 80% of VO_2_max [57]. Overall, it appears that interactions between intensity, duration, and type of exercise might affect exercise-induced prolactin responses [58,59,60]. In our study, both protocols had a mean intensity of 105% of AT (70%VO_2_max) and lasted for an hour, and the decline in the prolactin levels only at the end of the ALT exercise may suggest that this prolactin response is a result of the combination of the duration (which was longer than the usual HIIT protocols) and the intensity fluctuations of ALT exercise rather than of a prolonged exercise of constant submaximal intensity (Figure 4). More studies are needed to identify potential interactions between the ALT exercise duration and intensity and the acute responses of prolactin.

Our study has some limitations. Although the subjects consumed the same diet on the day before both ALT and CON exercise protocols and exercised after an overnight fast, the lack of a controlled diet for a widen period does not allow us to draw more stable and informative conclusions regarding the possible effects of diet on the acute glycemic and hormonal responses observed. In addition, the time of blood sampling during and after the exercise bouts may be a contributing factor for not revealing the representative timeline and the “window” of the largest hormonal responses to each exercise protocol. Also, we did not measure energy expenditure, fat oxidation, or other hormones, such as serotonin and dopamine, that could shed more light on the factors that regulate the (differential) hormonal responses to these two types of exercise. Lastly, our study population was healthy young, physically active males, so our findings cannot be generalized to other populations.

## 5. Conclusions

In this study, we found that cycling exercise of constant or alternating moderate intensity with the same duration induces similar glycemic but differential over time hormonal responses in healthy males. The systemic hormonal responses observed may point to their involvement in the homeostatic mechanisms activated during the exercise of different intensities though of the same duration and total work. It can be assumed that the hormones released into the circulation act as part of a regulatory network to support and control an effective response to different exercise demands. Further studies are required to better characterize the mechanisms by which hormonal responses are triggered and regulated at the systemic level during these types of exercise and recovery. Moreover, it would be of particular interest to investigate the end-organ effects of these hormonal alterations on target organs during exercise, thus revealing potential clinical implications of these types of exercise.

## Figures and Tables

**Table 1:** Anthropometric characteristics, maximum VO_2_ (VO_2_max), maximum power output (POmax), lactate threshold (LT), and heart rate maximum (HRmax) of the study participants. Values are presented as mean ± standard deviation (mean ± SD).

Age	Body Mass	Body Fat	Height	VO_2_max	POmax	LT	HRmax
(yrs)	(kg)	(%)	(cm)	(mL/kg/min)	(W)	(% of VO_2_max)	(b/min)
24.7 ± 4.7	78.5 ± 8.9	9.1 ± 3.1	180.8 ± 6	4 7.9 ± 6.5	266.8 ± 17.7	67.4 ± 3.6	188 ± 12

## Data Availability

All data generated or analyzed during this study are included in the article as Table(s) and Figure(s).

## Supplementary Materials

The following supporting information can be downloaded at: https://www.mdpi.com/article/10.3390/jcm14020457/s1, Table S1: Numeric data of the variables measured.
