# Supplementary material for: Hormonal and Glycemic Responses During and After Constant- and Alternating-Intensity Exercise
Source: J Clin Med. 2025 Jan 13;14(2):457. doi: 10.3390/jcm14020457 (PMC11765682; doi:10.3390/jcm14020457)
Supplement: Supplementary file 1 [file jcm-14-00457-s001.zip › jcm-3366177-supplementary.pdf]

Supplementary Table S1. Numeric data of the variables measured.

Glucose (mg/dl)

	ALT			
	**PRE**	**30 MIN EX**	**60 MIN EX**	**60 min Post- EX**
**MEAN**	87,80	82,69	89,21	85,04
**SE**	3,87	3,59	4,10	4,09

	CON			
	**PRE**	**30 MIN EX**	**60 MIN EX**	**60 min Post- EX**
**MEAN**	78,80	81,63	80,39	78,61
**SE**	4,06	3,46	5,94	2,19

Insulin (μIU/ml)

Significantly different compared to baseline, (PRE): ***p* < 0.01, # Significantly different compared to 60 min.

	ALT			
	**PRE**	**30 MIN EX**	**60 MIN EX**	**60 min Post- EX**
**MEAN**	19,73	15,48#	6,78**	14,76
**SE**	4,88	4,23	3,40	4,74

	CON			
	**PRE**	**30 MIN EX**	**60 MIN EX**	**60 min Post- EX**
**MEAN**	20,83	13,00*	7,78**	9,05***
**SE**	3,21	3,62	2,79	2,61

Significantly different compared to baseline, (PRE): **p* < 0.05; ***p* < 0.01, ****p* < 0.01

Leptin (ng/ml)

	ALT			
	**PRE**	**30 MIN EX**	**60 MIN EX**	**60 min Post- EX**
**MEAN**	1,36	1,38	0,86	1,21
**SE**	0,36	0,35	0,16	0,32

	CON			
	**PRE**	**30 MIN EX**	**60 MIN EX**	**60 min Post- EX**

**MEAN**	1,70	0,84*	0,71**	1,04
**SE**	0,39	0,16	0,15	0,23

Significantly different compared to baseline, (PRE): *$p < 0.05$; **$p < 0.01$.

Prolactin (ng/ml)

	**ALT**			
	**PRE**	**30 MIN EX**	**60 MIN EX**	**60 min Post- EX**
**MEAN**	2,20	1,78	1,30	1,86*
**SE**	0,28	0,33	0,20	0,27

	**CON**			
	**PRE**	**30 MIN EX**	**60 MIN EX**	**60 min Post- EX**
**MEAN**	1,94	1,57	1,52	1,61
**SE**	0,30	0,16	0,23	0,23

Prolactin levels were significantly decreased only at 60 min of ALT exercise compared to baseline (PRE), ($p < 0.05$).

ALT: Alternating-Intensity Exercise, **CON:** Constant Exercise

PRE: Baseline, **30 MIN EX, 60 MIN EX**: Exercise, **60 MIN post-EX:** Post-Exercise

SE: standard error of the mean
